# Is trigeminal neuralgia the only definitive diagnosis for pain in a tooth extraction site?

**DOI:** 10.1186/s12903-021-01645-6

**Published:** 2021-06-07

**Authors:** Arman Taheri, Shahram Sepehrmand

**Affiliations:** 1 Brain and Spinal Cord Injury Research Center, Neuroscience Institute, Tehran University of Medical Sciences, Tehran, Iran; 2 Department of Anesthesiology and Critical Care and Pain Medicine, IKHC Hospital, Tehran University of Medical Sciences, Tehran, Iran; 3 Honorary Consultant in Orofacial Pain Department, International Campus Dental School, Tehran University of Medical Sciences, Tehran, Iran

**Keywords:** Orofacial pain, Trigeminal neuralgia, Tooth extraction, Nerve injury, CRPS, Trigeminal neuropathy, Carbamazepine

## Abstract

Knowing the International Classification of Orofacial Pain helps pain specialists to differentiate types of orofacial pain. It is important to select the best treatment or intervention for the patients based on the diagnosis. As part of our study, we reviewed the article published in BMC Oral Health, titled “Clinical characteristics and associated factors of trigeminal neuralgia: Experience from Addis Ababa, Ethiopia” by Ayele et al. (Ethiopia BMC Oral Health 20(1):7, 2020). For patients suffering from Classical Trigeminal Neuralgia taking a suitable dose of Carbamazepine or Gasser Ganglion radiofrequency could be helpful. Patients complaining Trigeminal neuralgia who had a history of a dental extraction in the painful region should be categorized in other group as Complex Regional Pain Syndrome type 1, who need larger dose of carbamazepine with anticonvulsant or tricyclic agent drugs (e.g. pregabalin or doxepin) or intervention (PPG radiofrequency).

## Main text

Definitive diagnosis for facial pain is a complicated task, and requires expertise and meticulousness. Misdiagnosis can result in incorrect treatments. Especially incautious description of symptoms can negatively affect scientific conversations, training of physicians and patients’ treatments.

In their published article at BMC-Oral Health, Ayele et al. suggested that there was a significant association between higher doses of Carbamazepine and history of tooth extraction [1]. Therefore, the authors hypothesized that their study participants with a positive history of tooth extraction would require higher doses of Carbamazepine—to relieve their facial pain—compared to those with no history of tooth extraction [1-p.3]. In conclusion, the authors observed a higher proportion of dental extraction among their patients, hinting at the scale of delayed diagnoses and miss-diagnoses [1-p.6]. We argue that the interpretation of the results in Table 4 of Ayele et al. article is incomplete and we aim to demonstrate how the analysis can be improved.

## Discussion

[1] claim that they included 61 TN patients in the research study [1-p.3]; and outwardly they used International Classification of Headache Disorders 3th edition (ICHD-3) for diagnosis and symptoms [1-p.2]. However, this claim does not seem true for 2 reasons. First, the authors have referred to the Beta-version of the guidance, published in 2013 [1-p.7]. Second, more than half of their patients had been diagnosed with TN more than 5 years ago, and 11.5% had been diagnosed with TN more than 10 years ago [1-p.3]. Since the paper was published in 2020, these diagnoses could have not been according to the ICHD-3, published in 2018. Therefore, the inclusion criteria in this research study are not accurate. According to the Beta ICHD-3, Post traumatic trigeminal neuropathy (PTTN), is categorized as TN, and inaccurate inclusion criteria reduces the effect size of the research study.

Special care is required with examining symptoms and diagnosis of patients with facial pain caused by dental-related traumas. Some patients complain of constant burning, and irregular tingling and stabbing pain. In combination of trophic changes, edema, and redness, a possible diagnosis for this condition could be reflex sympathetic dystrophy. However, if patients report significant burning pain, a treatment such as sympathetic blockade of the stellate ganglion could be considered. [2-p.296]

Ayele et al. [1] mentioned in the background section that “quality of pain related to TN is described as electric shock-like, sharp, stabbing or shooting” [1-p.2]. However, in the abstract, their participants “reported mixed types of pain such as burning, lancinating, and electric shock-like” [1-p.1].

Table 4 of Ayele et al. article, showed that (10, 16.4%) of their patients had burning pain (quality of pain) and (13, 21.3%) of them had a feeling similar to being injected with a red hot needle. According to the International Classification of Headache Disorders (ICHD-3) (13.1.1) these two forms of pain quality are not included in diagnostic criteria of Trigeminal Neuralgia; meanwhile, the last criterion in diagnostic criteria of TN (D) is, “not better accounted for another ICHD-3 diagnosis” [3-p.166]. Furthermore, painful Trigeminal Neuropathy (13.1.2) is “described as burning or squeezing, or likened to pins and needles” [3-p.169]. Ayele et al. suggest that “the discrepancies in pain characterization could be explained by patients’ experience and response to an open or closed question which may lead the patient to unspecific answer” [1-p.6]. We are not convinced by this justification and believe this discrepancy needs to be examined more carefully. In addition, one of the diagnostic criteria mentioned in the ICHD-3 for painful post-traumatic trigeminal neuropathy (13.1.2.3 of ICHD-3) is that “pain is localized to the distribution(s) of the Trigeminal nerve(s) affected by the traumatic event” [3-p.170]. Hence, it is unjustified to claim that Trigeminal Neuralgia is the only diagnosis for pain in tooth extracted site(s). However, attribution of the requirement of higher doses of Carbamazepine for patients with a positive history of tooth extraction toward delayed or miss-diagnosis is reasonable and wise; but, it is not sufficient. These clues guide clinicians to other probable diagnoses. And each diagnosis will run separate treatments that are so different in outcomes [3].

### Diagnosis

A possible diagnosis for patients with unusual pain in a tooth extraction site will be PTTN that has different pain quality [3]. In more recent articles, persistent pain caused by Trigeminal nerve injury has also been named as Atypical Odontalgia (AO), phantom tooth pain, or atypical facial pain [4]. However, for some patients AO could arguably be termed as CRPS [5, 6]. Behrman (1949), Jaeger et al. (1986), Saxen and Campbell (1995) [as cited in 5] reported CRPS after tooth extraction from the molar region. Meanwhile, in order to International Classification of Orofacial Pain 1st edition (ICOP) many types of pain could be found in tooth extracted site(s) [7]. Therefore, selecting an exact name for the chosen criteria in the study of Ayele et al. [1] is unfeasible. Using names such as, Classical Trigeminal Neuralgia (CTN), Secondary Trigeminal Neuralgia (STN) or Idiopathic Trigeminal Neuralgia (ITN) for patients with pain in the tooth extraction site(s) is incorrect.

### Treatment

Trigeminal Neuralgia is usually responsive, at least initially, to pharmacotherapy (especially Carbamazepine) [4, 5]. Treatment of neuropathy is based on anti-neuropathic agents and drugs. Carbamazepine that is successful for (TN) is ineffective for (PTTN) [4]. Neurosurgical options that are useful for (TN), are contraindicated or unsuccessful in (PTTN) [4]. Radiofrequency treatment is an effective method for reducing pain in patients with pain caused by TN [8]; but to the best of our knowledge, no study can be found indicating usefulness of this method to Neuropathies.

## Conclusion

We believe that such a nonchalant approach to a complicated pain can lead to wrong diagnoses and consequently wrong treatment plans. Burning pain would be a clue to other diagnoses than TN.

## Data Availability

Not applicable.

## Abbreviations

CTN: Classical trigeminal neuralgia
TN: Trigeminal neuralgia
STN: Secondary trigeminal neuralgia
ITN: Idiopathic trigeminal neuralgia
ICHD-3: International classification of headache disorders-3rd edition
IHS: International headache society
PTTN: Post-traumatic trigeminal neuropathy
MS: Multiple sclerosis
AO: Atypical odontalgia
CRPS: Complex regional pain syndrome
ICOP: International classification of orofacial pain
TCA: Tricyclic antidepressants
PPG: Posterior palatine ganglion
